# Functional Alterations in Cerebellar Functional Connectivity in Anxiety Disorders

**DOI:** 10.1007/s12311-020-01213-8

**Published:** 2020-11-18

**Authors:** Yoon Ji Lee, Xavier Guell, Nicholas A. Hubbard, Viviana Siless, Isabelle R. Frosch, Mathias Goncalves, Nicole Lo, Atira Nair, Satrajit S. Ghosh, Stefan G. Hofmann, Randy P. Auerbach, Diego A. Pizzagalli, Anastasia Yendiki, John D.E. Gabrieli, Susan Whitfield-Gabrieli, Sheeba Arnold Anteraper

**Affiliations:** 1 Department of Psychology, ISEC 672D, Northeastern University, Boston, MA 02115 USA; 2 Massachusetts General Hospital, Boston, MA USA; 3 University of Nebraska-Lincoln, Lincoln, NE USA; 4 Massachusetts Institute of Technology, Cambridge, MA USA; 5 Athinoula A. Martinos Center for Biomedical Imaging, Massachusetts General Hospital, Boston, MA USA; 6 Harvard Medical School, Boston, MA USA; 7 Boston University, Boston, MA USA; 8 Columbia University, New York, NY USA; 9 McLean Hospital, Belmont, MA USA

**Keywords:** Anxiety, Cerebellum, Dentate nucleus, Salience network, Motor network

## Abstract

Adolescents with anxiety disorders exhibit excessive emotional and somatic arousal. Neuroimaging studies have shown abnormal cerebral cortical activation and connectivity in this patient population. The specific role of cerebellar output circuitry, specifically the dentate nuclei (DN), in adolescent anxiety disorders remains largely unexplored. Resting-state functional connectivity analyses have parcellated the DN, the major output nuclei of the cerebellum, into three functional territories (FTs) that include default-mode, salience-motor, and visual networks. The objective of this study was to understand whether FTs of the DN are implicated in adolescent anxiety disorders. Forty-one adolescents (mean age 15.19 ± 0.82, 26 females) with one or more anxiety disorders and 55 age- and gender-matched healthy controls completed resting-state fMRI scans and a self-report survey on anxiety symptoms. Seed-to-voxel functional connectivity analyses were performed using the FTs from DN parcellation. Brain connectivity metrics were then correlated with State-Trait Anxiety Inventory (STAI) measures within each group. Adolescents with an anxiety disorder showed significant hyperconnectivity between salience-motor DN FT and cerebral cortical salience-motor regions compared to controls. Salience-motor FT connectivity with cerebral cortical sensorimotor regions was significantly correlated with STAI-trait scores in HC (*R*^2^ = 0.41). Here, we report DN functional connectivity differences in adolescents diagnosed with anxiety, as well as in HC with variable degrees of anxiety traits. These observations highlight the relevance of DN as a potential clinical and sub-clinical marker of anxiety.

## Introduction

Anxiety disorders are the most common mental illnesses among adolescents, with the median age of onset at 11 years and a lifetime prevalence rate of 31.9% during adolescence [1, 2]. Adolescence is a unique developmental period during which sensitivity to affective information peaks and emotional responses to stimuli are particularly intense [3]. Adolescent anxiety disorders may thus lead to prolonged psychosocial problems and be a precursor to other psychiatric disorders such as major depressive disorders [4]. Neurocognitive studies on adolescent anxiety have mostly focused on amygdala and prefrontal cortex due to their roles in emotion- and cognitive control-related behaviors [5–7]. However, anxiety disorders are associated with widespread network disruption [8].

Anxiety disorders are characterized by the exaggerated aversive response to actual or perceived threatening stimuli, often accompanied by emotional, cognitive, and somatic arousal [9]. One region implicated in motor, cognitive, and emotional processes involved in anxiety disorders is the cerebellum. Neuroimaging studies have highlighted cerebellar changes associated with anxiety disorders. Specifically, increased cerebellar gray matter volume has been reported in social anxiety disorder [10] and specific phobia [11]. Relative to healthy participants, patients with anxiety disorders have shown enhanced cerebellar activity when presented with angry faces [12], social tasks [13], and at rest [14]. Patients with anxiety disorders also display changes in cerebellar functional connectivity with anxiety-related regions in the cerebral cortex, such as the limbic system and prefrontal cortical areas. For example, enhanced connectivity between cerebellum and amygdala, a core structure implicated in emotion processing, has been reported in multiple studies of generalized anxiety disorder [5, 7, 15]. Furthermore, in patients with anxiety disorders, the cerebellum has shown aberrant intrinsic connectivity with the salience network [16, 17], default-mode network (DMN) [18], and central-executive networks (Hilber et al.) [19] during resting-state functional magnetic resonance imaging (fMRI). Such alterations in cerebellar connectivity are also evident in non-clinical populations with high state or trait anxiety [20, 21], suggesting that the cerebellum has a role in anxiety susceptibility.

**Fig. 1:**
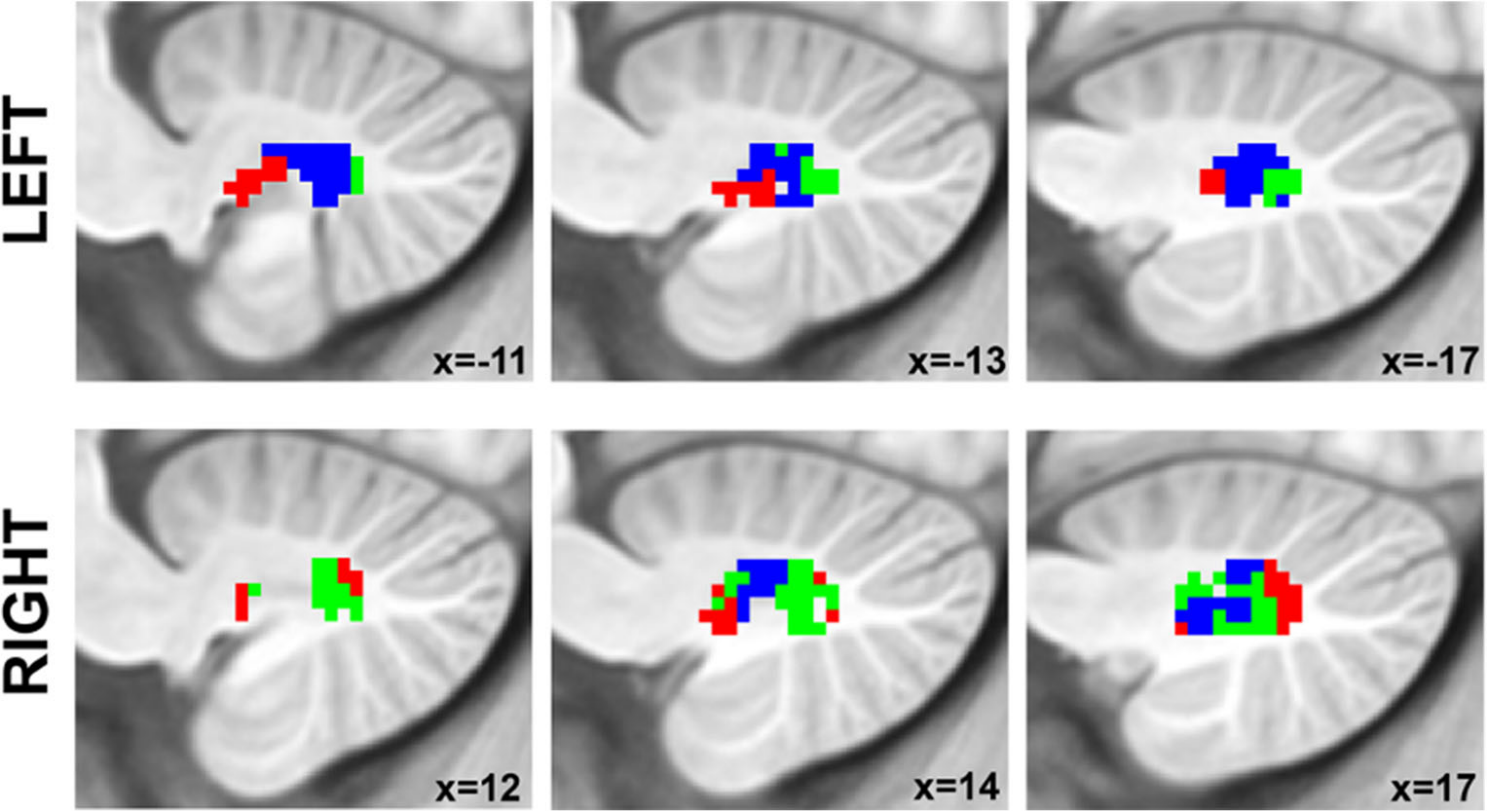
Structural location and FTs of the dentate nuclei as reported in [26]). Red, FT1 = default-mode network FT. Blue, FT2 = salience-motor FT. Green, FT3 = visual FT

Although a cerebellar role in anxiety disorders has been evidenced in the literature, prior investigation has rarely examined abnormalities in the cortico-cerebellar functional networks of adolescents with anxiety disorders. The cerebellum communicates with cortical regions by projecting to the deep cerebellar nuclei, the largest of which is the dentate nuclei (DN). DN projects first synapse in the thalamus, and then projects to frontal, motor, and parietal cortices, allowing the cerebellum to contribute to virtually all streams of information processing in the cerebral cortex [22, 23]. As the cerebellum serves a wide range of functions and is suggested to be composed of discrete regions dedicated to unique functions [24, 25], we aimed to identify whether alterations in cerebellar functional connectivity in adolescent anxiety are predominantly located within a specific functional territory within the DN or whether these alterations are present in all aspects of the DN. Here, we employed the functional parcellations of DN, the major cerebellar output nuclei, from Guell et al.’s [26] research, which parcellated the DN into three territories with specific functional connections to default-mode, salience-motor, and visual networks in the cerebral cortex using resting-state fMRI scans in a healthy population. We used these three DN functional territories (FTs) as seed regions of interest (Fig. 1) to newly examine the intrinsic functional connectivity of cortico-cerebellar network in adolescent anxiety disorders and investigate its relationship with individual differences in anxious symptoms.

## Methods

### Study Participants and Procedure

Adolescents aged 14–17 diagnosed with anxiety disorders (Anx = 41; mean age = 15.19) and matched healthy controls (HC = 55, mean age = 15.31) were enrolled in the Boston Adolescent Neuroimaging of Depression and Anxiety (BANDA) project. Clinical characteristics, diagnostic criteria, and demographics information have been previously reported (see [27, 28]). Sample clinical characteristics are summarized in Table 1.

**Table 1:** Demographics

	Anx	HC	*p* value
Demographics			
Total participants	41	55	
Mean age (years)	15.19 ± 0.82	15.31 ± 0.86	*p* = 0.51
Sex: female	26 (63)	31 (56)	
Handedness: right-handed	39 (95)	46 (83)	
Full-scale WASI score	115.80 ± 16.40	118.13 ± 14.11	*p* = 0.47
STAI: trait score	43.98 ± 9.67	30.45 ± 7.44	*p* < 0.001
STAI: state score	39.4 ± 10.49	29.18 ± 8.04	*p* < 0.001
Psychotropic medication treatment	18 (44)	0	
Quality assurance			
No. of invalid scans	123.88	109.11	*p* = 0.45
Max motion	0.69	0.53	*p* = 0.37
Mean motion	0.06	0.06	*p* = 0.98
Anxiety disorders			
Generalized anxiety disorder	23 (56)	0	
Social anxiety disorder	18 (44)	0	
Overanxious disorder	17 (41)	0	
ADHD	11 (27)	0	
Specific phobia	9 (22)	0	
Oppositional defiant disorder	5 (12)	0	
OCD/excoriation disorder	5 (12)	0	
Separation anxiety	4 (10)	0	
Avoidant personality disorder	4 (10)	0	
Panic disorder	4 (10)	0	
Substance use disorder	1 (2.4)	0	
PTSD	1 (2.4)	0	
Others	4 (10)	0	
Types of medication treatment			
Serotonin reuptake inhibitor (SSRI)	14 (78)	0	
Stimulant	4 (22)	0	
Antipsychotic drug	1 (6)	0	
Benzodiazepine	1 (6)	0	
Tricyclic antidepressant	1 (6)	0	
Anxiolytic agent	1 (6)	0	
Alpha-agonist agent	1 (6)	0	
Anticonvulsant	1 (6)	0	

Values expressed as *n* (%) or mean ± standard deviation; *WASI*, Wechsler Abbreviated Scale of Intelligence; *ADHD*, attention-deficit/hyperactivity disorder; *OCD*, obsessive-compulsive disorder; *PTSD*, post-traumatic stress disorder; *Others* include Tourette syndrome, enuresis, misophonia, and unspecified anxiety disorder

Informed consent was obtained from legal guardians and assent was obtained from the adolescent. Adolescent-parent dyads were administered the KSADS to assess adolescent lifetime mental disorders, and the State-Trait Anxiety Inventory (STAI; [29]) was used to assess continuous anxiety. The STAI is a commonly used self-report questionnaire that measures both anxiety levels rooted in the personality (STAI-trait) and anxiety as a transitional emotional state (STAI-state); each subscale ranges from 20 to 80, and higher scores indicate a greater level of anxiety [30].

### Structural and Resting-State Functional MRI Acquisition Parameters

Imaging data were collected on a Siemens 3T Prisma whole-body scanner with vendor-provided 64-channel head coil (Siemens Healthcare, Erlangen, Germany). High-resolution structural data (0.8-mm isotropic voxels) were acquired using a T1-weighted MPRAGE sequence with a duration of 7 min 50 s (in-plane acceleration factor of 2). Scan parameters for TR, TE, TI, and flip angle were 2.4 s, 2.18 ms, 1.04 s, and 8°. Anatomical scans were immediately followed by resting-state scans, during which subjects were asked to stay awake and keep their eyes fixated on a crosshair. Four resting-state sessions per participant were acquired, with two scans of diffusion MRI in AP-PA directions in the middle. Scan parameters (T2*-weighted EPI sequence) for TR, TE, flip angle, echo spacing, and bandwidth were 800 ms, 37 ms, 85°, 0.58 ms, and 2290 Hz per pixel. Seventy-two interleaved (ascending/foot-head) slices were collected in the AC-PC plane using an auto-align procedure to minimize inter-subject variability in data acquisition. Combination of 64ch array coil and simultaneous multi-slice (SMS) acquisition (multiband factor of 8) provided high temporal sampling (420 time points during an acquisition window of 5 min and 46 s; four runs) and spatial resolution (2 mm isotropic) while maintaining whole-brain coverage (including the entire cerebellum).

### Data Processing: Seed-to-Voxel Functional Connectivity Analysis

Resting-state data were realigned and spatially normalized to the MNI template using SPM12 (Wellcome Department of Imaging Neuroscience; www.fil.ion.ucl.ac.uk/spm). Structural images were segmented into white matter (WM), gray matter, and cerebrospinal fluid (CSF) using SPM12. The CONN Toolbox [31] was used to compute whole-brain correlation maps from the seed regions of interest (ROIs). ROIs included the whole DN (as defined using the SUIT DN mask [32]), and three functional sub-territories of DN that were defined in a previous study by our group [26], including default-mode network, motor-salience, and visual functional regions (see Fig. 1). The CONN Toolbox uses an anatomical component-based correction method (aCompCor [33]) for denoising BOLD time series and integrates quality assurance (QA) methods to address the deleterious effects of motion artifacts (Artifact Detection Tools, www.nitrc.org/projects/artifact_detect). There was no between-group difference in the number of motion outliers and maximum and mean head motion. Band-pass filtering was carried out at 0.008–0.09 Hz. Time points with a mean signal intensity beyond three standard deviations from the global mean signal and 0.4-mm scan-to-scan motion (about one-fifth the acquisition voxel size) were flagged as problematic scans and were regressed out along with six realignment parameters (along with derivatives) and physiological sources of noise (three principal components of WM, and three principal components of CSF segments, using aCompCor [33]). WM and CSF segments were derived from the structural images using the segmentation routine in SPM12. Because of the small size of the DN, unsmoothed data were used for data analysis to minimize partial volume effects from structures close to DN [34]. Whole-brain Pearson’s correlation maps derived from denoised time series from whole DN and the three DN functional territories were converted to *z*-scores using Fisher’s *r* to *z* transformation to carry out second-level general linear model (GLM) analyses.

### Data Processing: Second-Level GLM Analysis

Seed-to-voxel analysis was carried out using the whole DN as a seed, as well as using the unique effect of each of the three functional territories (DMN, salience-motor, and visual). The unique effect of each functional territory [35] was calculated using a previously described method [26]. Specifically, the DMN unique effect (FT1) was calculated as DMN > (salience-motor and visual), salience-motor unique effect (FT2) was calculated as salience-motor > (DMN and visual), and visual unique effect (FT3) was calculated as visual > (DMN and salience-motor). Statistical significance thresholding for between-group effects included *p* < 0.001 (two-sided) at the voxel level and *p* < 0.05 false discovery rate (FDR) correction at the cluster level.

### Symptom Correlation Analyses

For all three functional territories, correlations between resting-state fMRI correlations and STAI scores were calculated in both HC and Anx groups, with a voxel threshold of *p* < 0.001 and a cluster-forming threshold of *p* < 0.05 (FDR corrected).

## Results

### Second-Level GLM Analysis

**Fig. 2:**
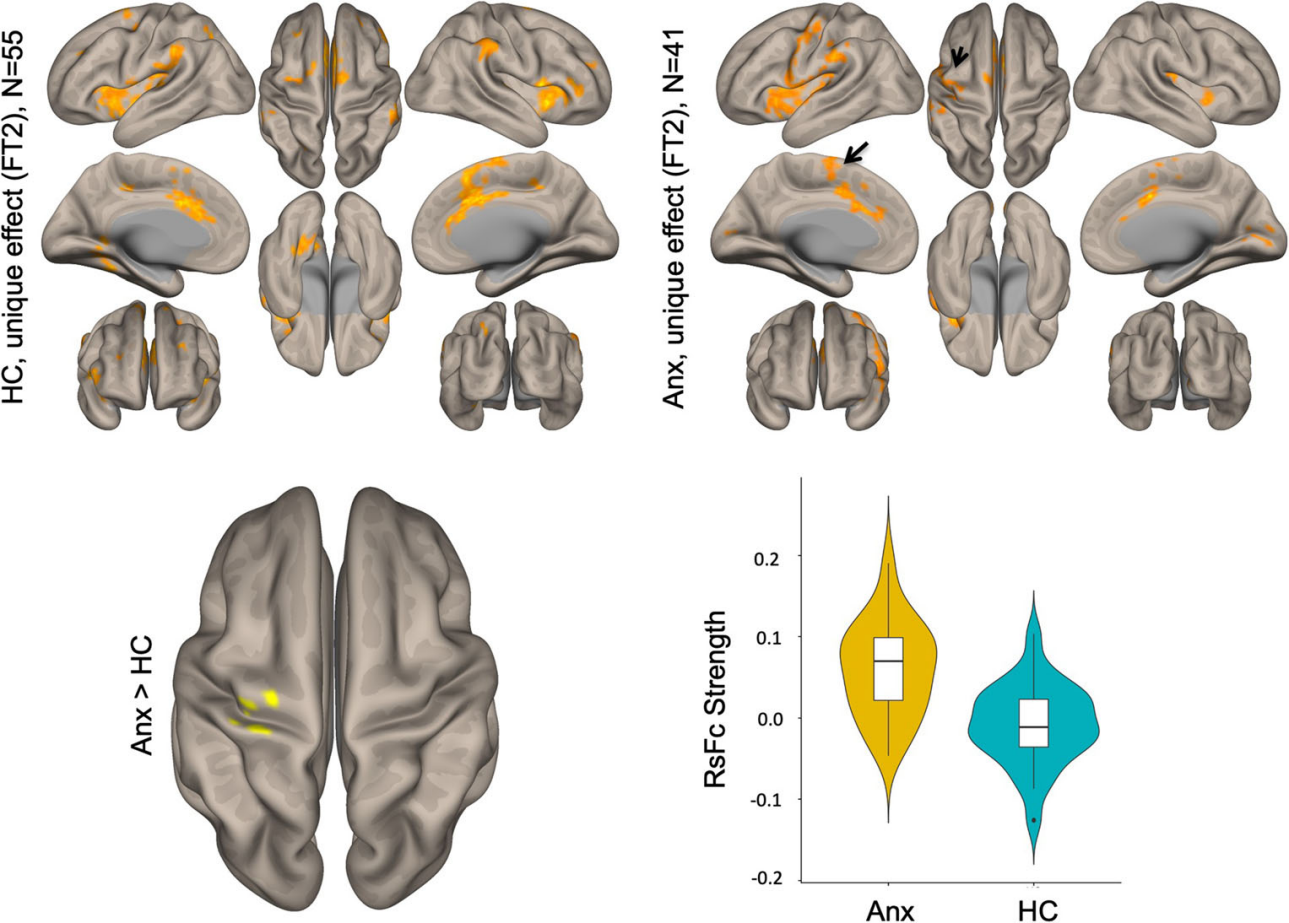
Top: Within-group results (overlaid on surface maps in CONN) using functional connectivity calculated from the salience-motor FT of the DN (FT2), at voxel-level height threshold of *p* < 0.001 (two-sided) and cluster size FDR correction of *p* < 0.05. Bottom: Between-group results after controlling for medication use (Anx > HC) at voxel-level height threshold of *p* < 0.001 (two-sided) and cluster size FDR correction of *p* < 0.05, *T* = 5.15. Bar plots provide data for the significant cluster (precentral and postcentral cerebral cortex) in Anx and HC

Within functional sub-regions of DN (see Fig. 1), statistically significant differences were detected only for the salience-motor territory (FT2), revealing increased functional connectivity with pre- and postcentral cerebral cortices (peak coordinate at (− 34, − 28, 70)) (Fig. 2). For both HC and Anx groups, within-group seed-to-voxel functional connectivity analyses for FT2 (Fig. 1, top panel) showed functional connectivity to cerebral cortical salience-motor regions including the primary motor cortex and supplementary motor area, as well as the bilateral insula, dorsal anterior cingulate cortex, anterior supramarginal gyrus, and rostral middle frontal gyrus. The results were significant after covarying for medication use. Using the whole DN as a seed did not reveal any statistically significant differences between HC and Anx, highlighting the relevance for investigating the functional parcellations of DN.

### Symptom Correlation Analyses

**Fig. 3:**
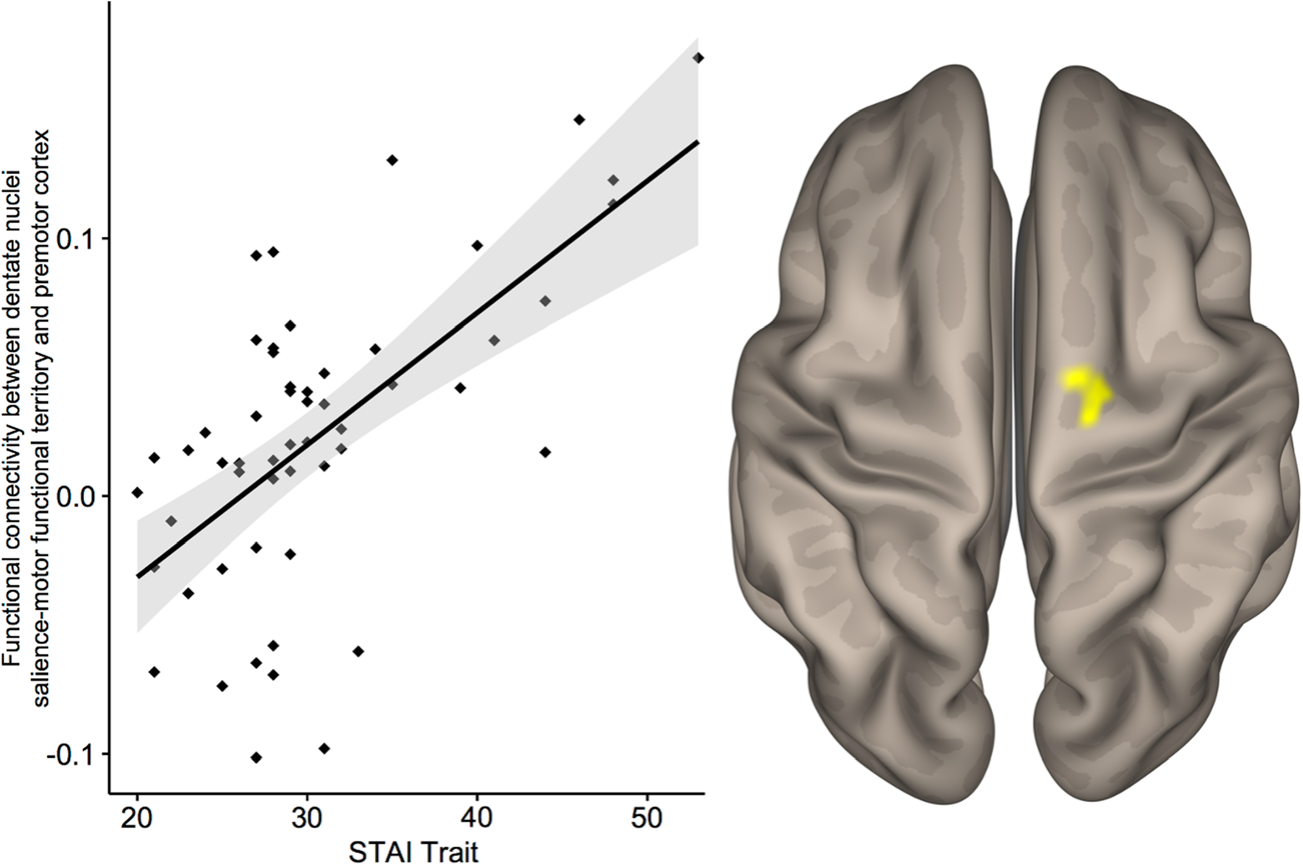
Whole-brain correlation between STAI-trait scores in healthy controls and salience-motor DN FT-motor/somatosensory cortex functional connectivity, thresholded at a height threshold of *p* < 0.001, cluster-corrected at *p* < 0.05 FDR. *R*^2^ = 0.41

To examine individual differences within each group, correlation analyses between functional connectivity and STAI scores were conducted separately for HC and Anx groups. Within HC, STAI-trait scores and functional connectivity between the salience-motor DN FC and cerebral motor/somatosensory cortex (20, − 8, 70) showed a significant correlation, linking higher trait anxiety level to stronger connectivity between salience-motor DN FT and cerebral cortical motor areas (Fig. 3). There was no significant cluster with STAI-trait scores within the Anx group. STAI-state scores did not reveal any significant difference in either group.

## Discussion

We show for the first time that functional connectivity alterations between cerebellar output structures and cerebral cortical areas are associated with adolescent anxiety. Specifically, hyperconnectivity was detected between DN salience-motor FT and primary motor and somatosensory cortices in adolescents with anxiety disorders compared to HC. No anxiety-related differences were observed with default mode or visual territories or all combined territories of the DN. Within HC, stronger functional connectivity of salience-motor DN to motor/somatosensory cortex correlated with higher trait anxiety. Taken together, this new evidence illustrates the use of DN functional sub-divisions as a relevant structure to detect functional differences in psychiatric disease and highlights the role of the DN as a potential target for disease prediction or prevention of affective disorders.

### Cerebellar-Cortical Salience-Motor Network in Anxiety

Evidence from both human and animal studies has established that the cerebellum is connected with various parts of anxiety circuitry, including the insula, basal ganglia, and ventral tegmental area (VTA) (see [8]) for review). Cerebellar connections with cortical and subcortical areas relevant in sensorimotor perception and anticipation of stimulus underpin the non-motor role of the cerebellum in anxiety. The cerebellar vermis, which contains the fastigial and dentate nuclei, is referred to as the “limbic cerebellum” for its connections to the mesolimbic dopaminergic pathway that originate from the VTA to the nucleus accumbens [36]. The dentate nucleus forms direct monosynaptic excitatory connections with neurons in the VTA [37], which is one of the key subcortical structures that is activated as part of the salience network [38]. Then, the dopaminergic fibers of the meso-cortico-limbic system project to motor cortical fields, mainly to the pre- and postcentral gyrus [39]. Hypoactivations in the cerebellar vermis and subcortical regions in the dopaminergic pathways have been associated with disturbed predictive motor timing paradigm [36].

Also, the insula sends efferent projections to the sensorimotor cortex from which it receives reciprocal afferent projections, forming the salience-motor network [40, 41]. Neuroimaging and invasive stimulation studies in humans support a physiological coupling between sensorimotor systems and stimulus-driven attentional processes [38, 40–44].

The cerebellum also receives inputs from the subthalamic nucleus of the basal ganglia, indicating that the cerebellum may be a key substrate for reward-related signals during learning [45]. Reward-based communication of the basal ganglia and the cerebellum has been highlighted during predictive motor timing tasks [46]. The salience-motor network plays an important role in detecting behaviorally relevant stimuli by mediating the switch between the DMN and task-positive central-executive network and permitting response to the stimuli [47]. The hyperconnectivity that we report in the DN salience-motor FT points to the possibility that there is a maladaptive attribution of salience to internal and environmental stimuli in anxious individuals [48]. It can also be interpreted as the discrepancy in the “internal model” of the cerebellar system that false internal representation of external stimuli would generate unsuited sensorimotor response, which leads to physiological stress and anxiety state [49].

Besides anatomical investigations, other studies of cerebellar function also support a role of the cerebellum in fear and anxiety in animals and humans. In rodent models, manipulations on cerebellar functioning impaired anxiety-related behaviors [50, 51]. Others have shown a cerebellar role in salience attribution to avoid harmful stimuli [14, 16, 52]. Patients with anxiety disorders also show changes in cerebellar activity and connectivity with anxiety-related brain areas. A few studies observed increased cerebellar activation in social anxiety disorder [12, 13] and specific phobia [53, 54]. Enhanced connectivity between the cerebellum and amygdala has been replicated several times across age range and symptom severity [5, 7, 15].

Functional connectivity differences between adolescents with anxiety disorders and HC were observed specifically in the salience-motor territory of the DN; the salience network has clear associations with fear and anxiety processing and is connected to sensorimotor processing systems in the brain as discussed earlier. The salience network is implicated in detecting emotional salience and triggering cognitive regulation [38]. Individuals with high trait anxiety or anxiety disorders have shown aberrance in the salience network. Weaker within-network salience connectivity has been associated with adolescents with higher trait anxiety and patients with social anxiety disorder, suggesting impaired ability in emotion regulation and overreaction to threatening stimuli [20, 55, 56]. One study on healthy young adults suggested that differential resting-state connectivity between the cerebellum and executive and salience cortical regions correlated with behavioral inhibition, which may have a mediating role in anxiety vulnerability [19].

### Symptom Correlation

Within the group of HC, STAI-trait scores correlated with functional connectivity between the premotor cortex and the salience-motor FT of the DN (Fig. 2). The somatosensory cortex is involved in emotional processing through its connection to the amygdala through the insula [57, 58]. After early evaluations of emotional significance are conducted through amygdala interaction with thalamus and limbic regions, somatosensory and related cerebral cortical areas take in for higher order re-evaluation of emotional perception to establish emotional salience [59] and generate fear memory [60]. Thus, people with higher alertness and sensitivity to stress stimuli may bring in more involvement of the somatosensory cortex displayed as heightened activity. It has also been reported that people with specific phobia show higher activation in the somatosensory cortex [61], and heightened activity in this area may contribute to difficulties in emotion regulation and lower self-control [62, 63]. Trait anxiety does not necessarily predict conversion to anxiety disorders but is an index of vulnerability to anxiety disorders. Individuals with high trait anxiety and those diagnosed with anxiety disorders share common disruptions in brain activation and connectivity [64, 65]. Individual differences in state anxiety, although not detected in this study, have also been correlated with increased fluctuations of activation in the right postcentral gyrus and right precentral gyrus and with connectivity between the postcentral gyrus and left cerebellum gyrus [21, 66]. The relationship between DN salience-motor FT connectivity and anxious symptoms may be shown in other populations with anxious symptoms [67].

### Limitations and Future Directions

First, our findings are based on exploratory analyses of cerebellar output structure that suggest overarching alterations in the cortical-cerebellar salience-motor network but do not specify the direction of connectivity. While DN functional connectivity may not uniquely correspond to unidirectional communication between the cerebellar cortex and extracerebellar structures, DN is the largest location of synapses for anatomical connections linking the cerebellar cortex to the thalamus and ultimately to the cerebral cortex. It is therefore reasonable to consider that DN functional connectivity is predominantly a measure of cerebellar functional output. Since we have understanding of anatomical connections between the cerebellum and cortical and subcortical regions of the salience-motor network, future studies can use causal modeling to characterize the effective connectivity between those regions and examine the directionality of the communication within the cortical-subcortical-cerebellar network.

Second, 44% of our Anx group were taking psychotropic medications. To overcome this limitation, we covaried for medication use in the between-group analyses. The number of adolescents not on medication use was small (*n* = 18), and STAI score correlation calculations within this subsample would have resulted in a substantial loss of statistical power making false-negative findings very likely; STAI score correlation analyses were thus performed on the full sample of the Anx group. We did not have information on behavioral therapy and thus could not control for this variable. The impact of behavioral therapy on the brain’s functional connectivity could have blurred individual differences in correlation between brain networks and anxiety symptoms within Anx as well. More generally, our findings provide only correlational evidence to support altered DN functional connectivity in anxiety disorders. Interventional studies such as non-invasive stimulation experiments or lesion-based analyses may establish a causal link between anxiety and alterations in DN functional connectivity.

## Conclusion

These findings advance our understanding of the cerebellar-cortical salience-motor network in anxiety disorders by identifying abnormal functional connectivity of salience-motor territories within DN, a major cerebellar output to the cerebral cortex, in adolescents with anxiety disorders. In addition, our study identified a relationship between trait anxiety level and DN salience-motor FT functional connectivity in adolescents without a diagnosis of anxiety disorder, suggesting DN salience-motor FT as a potential biomarker for abnormal emotional and attentional processing in sub-clinical anxiety. With recent advances in the field of neuromodulation and neurostimulation, these findings also illustrate the idea that the salience-motor territories in DN may be used as an experimental target region for non-invasive neuromodulation for treatment or prevention of anxiety disorders in the near future.
